# Surgical Treatment of Periprosthetic Acetabular Fractures After Hip Arthroplasty: A Report of Two Cases

**DOI:** 10.7759/cureus.82890

**Published:** 2025-04-24

**Authors:** Masahiro Matsumoto, Hyonmin Choe, Naomi Kobayashi, Ichiro Takeuchi, Yutaka Inaba

**Affiliations:** 1 Advanced Critical Care and Emergency Center, Yokohama City University Medical Center, Yokohama, JPN; 2 Orthopaedic Surgery, Yokohama City University School of Medicine, Yokohama, JPN; 3 Orthopaedic Surgery, Yokohama City University Medical Center, Yokohama, JPN

**Keywords:** bipolar hip arthroplasty (bha), della valle classification, paprosky classification, periprosthetic acetabular fracture, periprosthetic fracture, total hip arthroplasty (tha)

## Abstract

Bipolar hemiarthroplasty (BHA) and total hip arthroplasty (THA) are standard treatments for hip disorders in the elderly. However, a rare postoperative complication known as periprosthetic acetabular fracture (PPAF) can occur, potentially affecting hip joint stability and implant longevity. The management of PPAF is guided by the Della Valle and Paprosky classifications, which assess implant stability and the extent of bone loss. In this study, we report two cases of PPAF occurring after BHA and THA. In Case 1, early open reduction and internal fixation were performed, successfully avoiding stem revision and achieving a favorable clinical outcome. In contrast, Case 2 was initially managed conservatively; however, due to fracture displacement progression and subsequent reinjury from a fall, surgical intervention became necessary, requiring acetabular reconstruction and implant revision. Treatment options for PPAF include conservative management, plate fixation, and implant revision. In many cases, early surgical intervention yields better outcomes. Therefore, close collaboration between orthopedic trauma surgeons and hip reconstruction specialists is essential. Given the limited number of reported cases, further accumulation of case data and evaluation of long-term outcomes are needed. Early classification and appropriate management are crucial for optimal treatment of PPAF.

## Introduction

Bipolar hip arthroplasty (BHA) and total hip arthroplasty (THA) are widely used as standard treatments for femoral neck fractures and osteoarthritis of the hip in elderly patients [1]. These procedures have been shown to provide pain relief, improve the range of motion, and restore functional ability. However, periprosthetic fractures have emerged as one of the challenging postoperative complications [2].

Periprosthetic acetabular fractures (PPAFs) are rare complications following BHA or THA. Although infrequent, these fractures can significantly affect joint stability and the longevity of the implant [3]. Therefore, treatment strategies for PPAFs should be carefully planned based on implant stability and bone defects using a reliable classification system. The modified Paprosky and Della Valle classification is currently the most widely adopted system, covering all known variants of PPAF and offering guidance for both surgical and non-surgical management [2-4].

This report presents two cases of PPAF occurring after BHA and THA. Each case was managed surgically, but the clinical courses and outcomes differed. Through these cases, we discuss the clinical strategies for managing PPAF.

## Case presentation

Case 1

**Figure 1:**
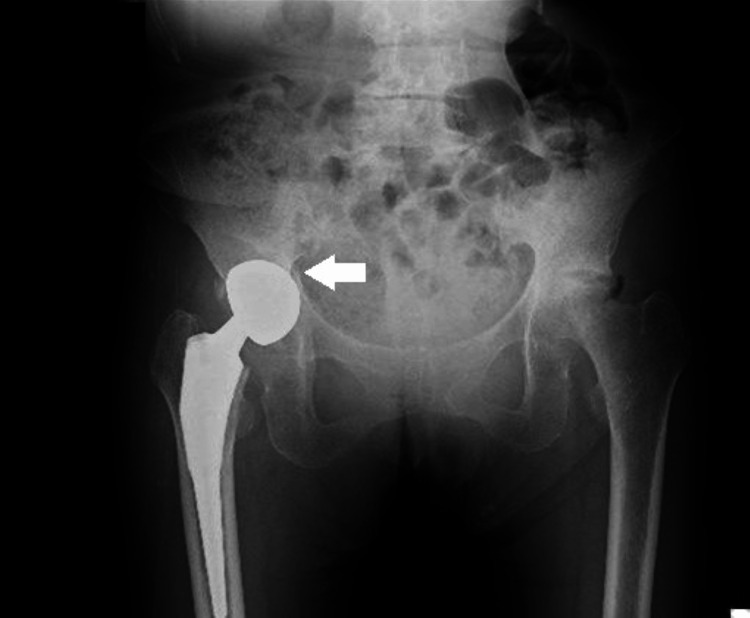
Plain radiograph of the pelvis at admission (anteroposterior view). A right acetabular fracture was identified (arrow), with no obvious loosening of the femoral stem.

**Figure 2:**
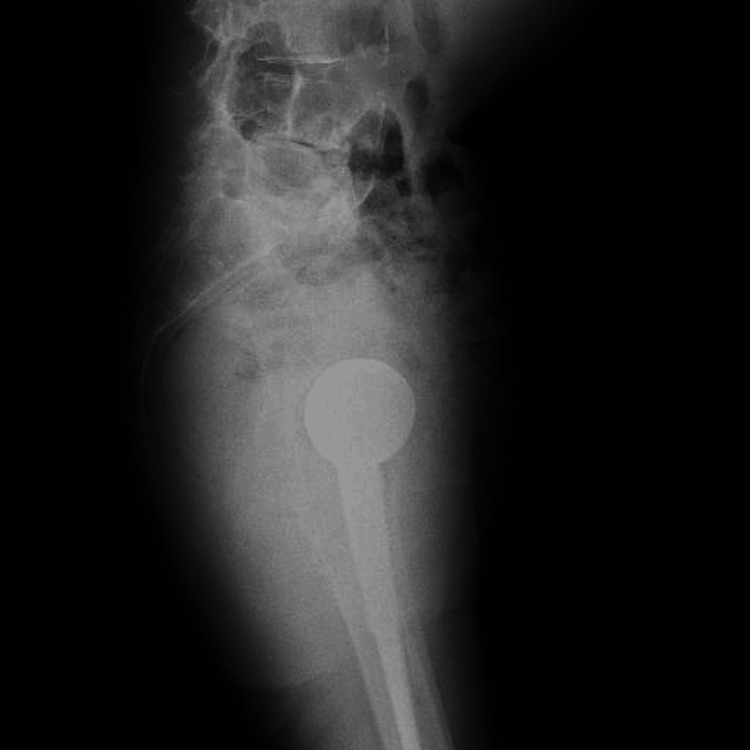
Plain radiograph of the pelvis at admission (lateral view). A right acetabular fracture was identified, with no obvious loosening of the femoral stem.

**Figure 3:**
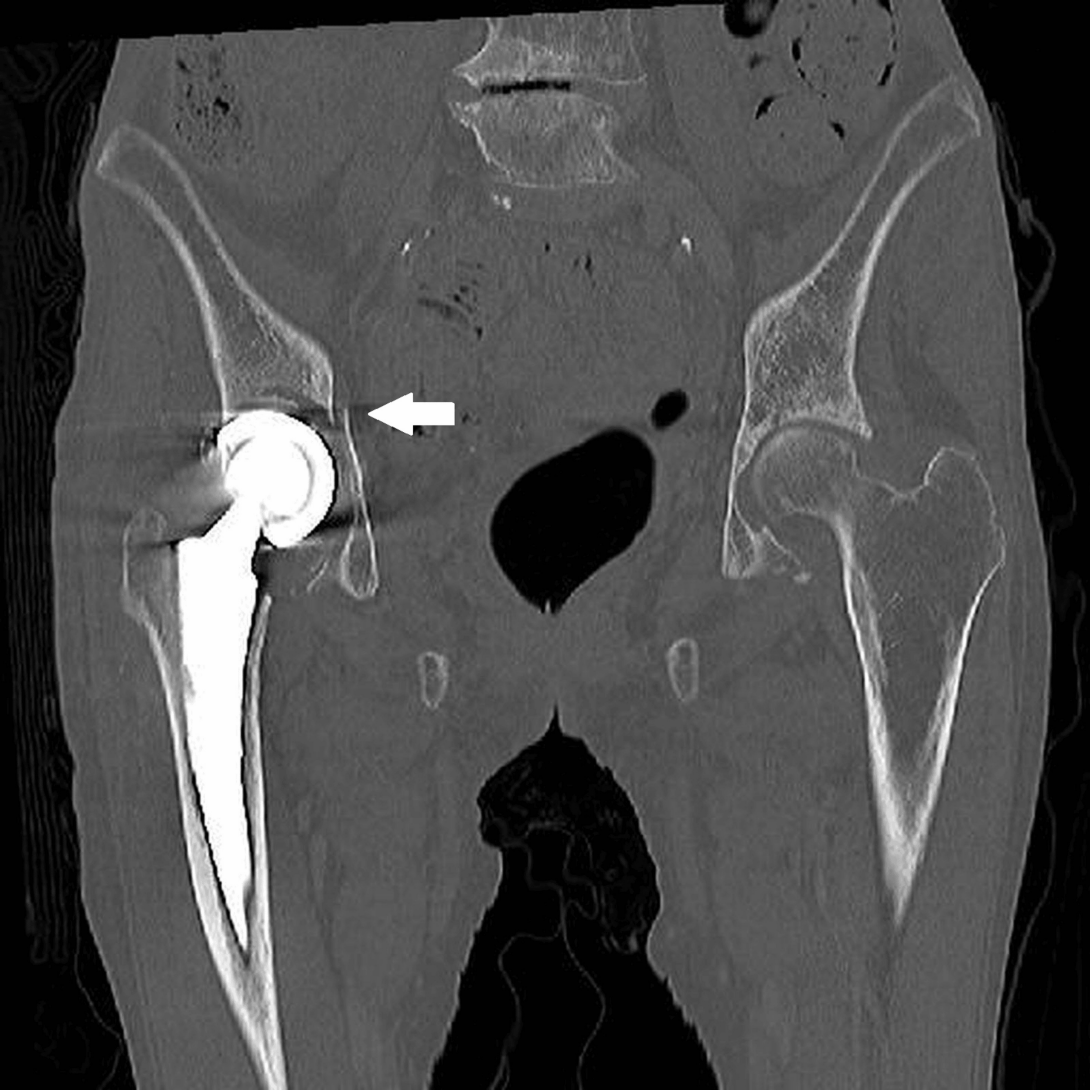
Pelvic CT coronal image on admission. A right acetabular fracture was identified (arrow), with no obvious loosening of the femoral stem.

**Figure 4:**
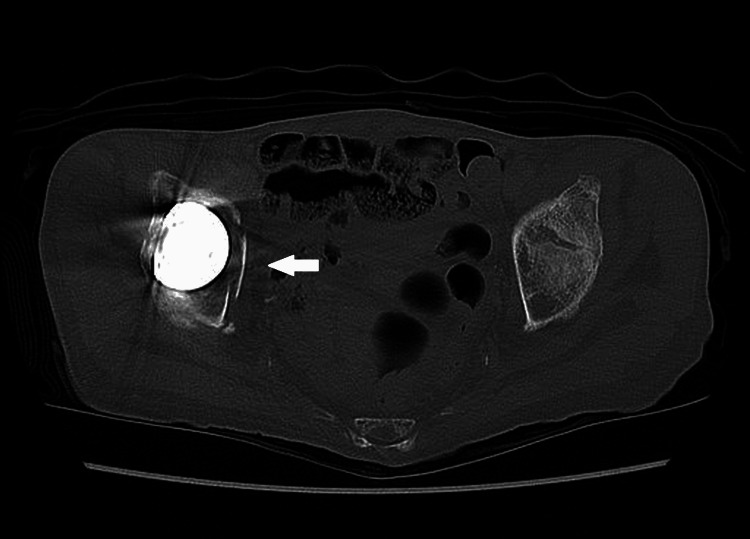
Pelvic CT axial image on admission. A transverse acetabular fracture was suspected based on findings from CT scans (arrow).

A 68-year-old woman presented with right hip pain following a fall. Her medical history included rheumatoid arthritis treated with methotrexate (12 mg/day). She had undergone BHA for a femoral neck fracture six months earlier. Imaging studies revealed fractures of the transverse of the acetabulum (Figures 1-4). Given the severe pain and high risk of dislocation, surgical intervention was planned.

**Figure 5:**
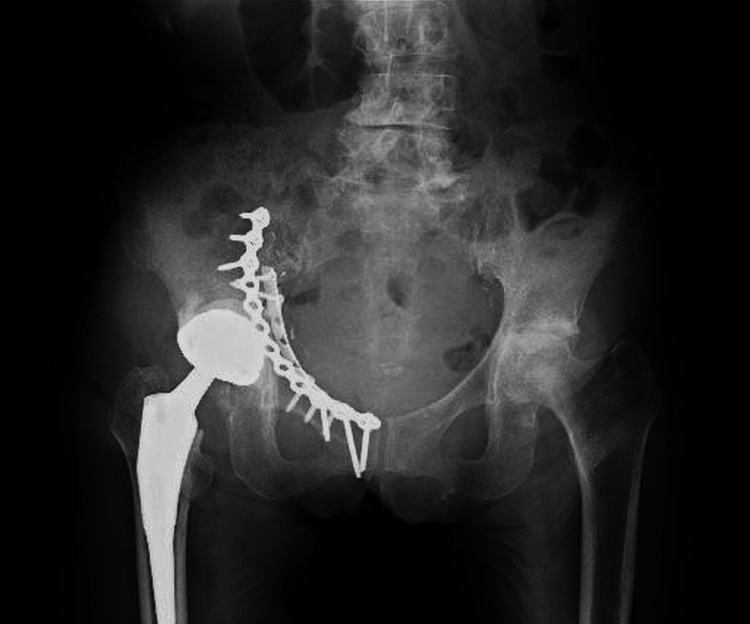
Postoperative plain radiograph of the pelvis (anteroposterior view). The fracture was stabilized using two locking plates placed anteriorly, and the acetabular component was revised with a cemented cup.

**Figure 6:**
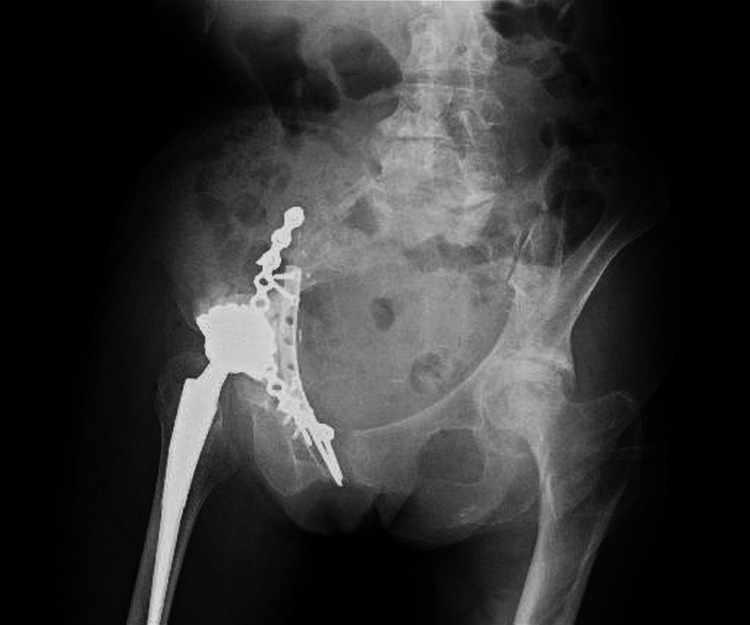
Postoperative plain radiograph of the pelvis (oblique view). The fracture was stabilized using two locking plates placed anteriorly, and the acetabular component was revised with a cemented cup.

Surgery for the acetabular fracture was performed in the supine position using a modified Stoppa approach to achieve open reduction and internal fixation (ORIF) with a locking plate. Subsequently, in the lateral decubitus position, the hip was re-approached using a direct lateral approach, and revision of the acetabular component of the total hip arthroplasty was performed. A cemented acetabular cup was implanted to ensure stable fixation in the setting of severe osteoporosis and revision surgery, and the femoral head was replaced. As the femoral stem remained stable, stem revision was not required (Figures 5, 6).

Postoperative rehabilitation began with non-weight-bearing (NWB) ambulation the day after surgery. Partial weight-bearing (PWB) was initiated at four weeks, and independent ambulation was achieved at three months.

Case 2

**Figure 7:**
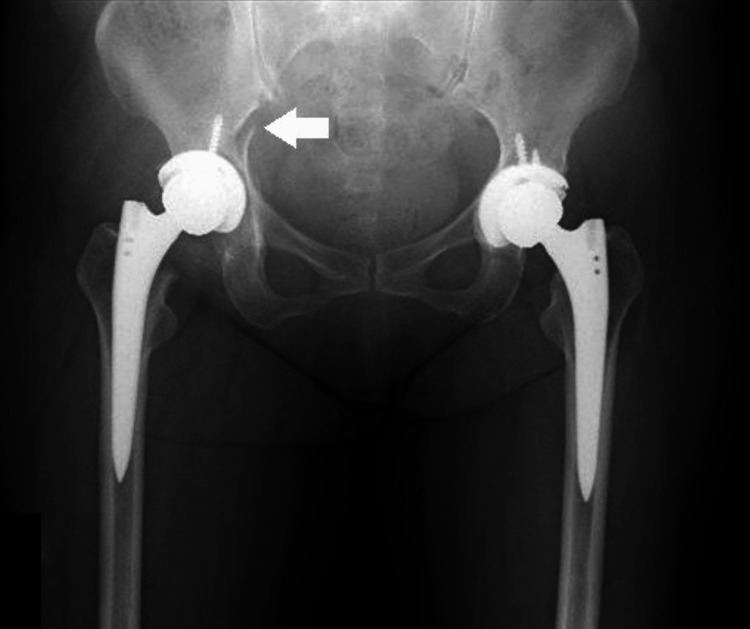
Plain radiograph of the pelvis at initial presentation (anteroposterior view). A right acetabular fracture was observed (arrow). There was no clear evidence of implant loosening.

**Figure 8:**
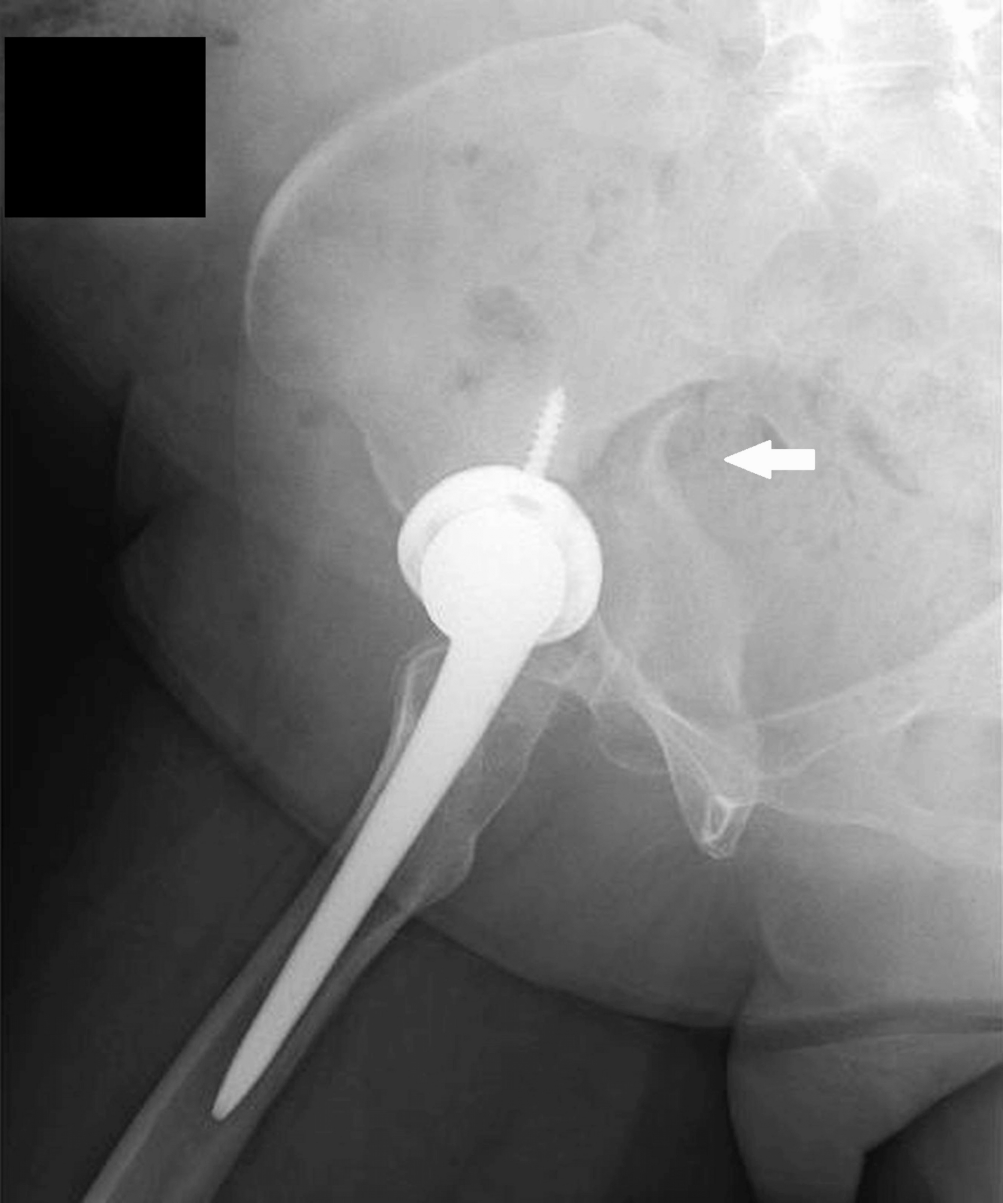
Plain radiograph of the pelvis at initial presentation (lateral view). A right acetabular fracture was observed (arrow). There was no clear evidence of implant loosening.

**Figure 9:**
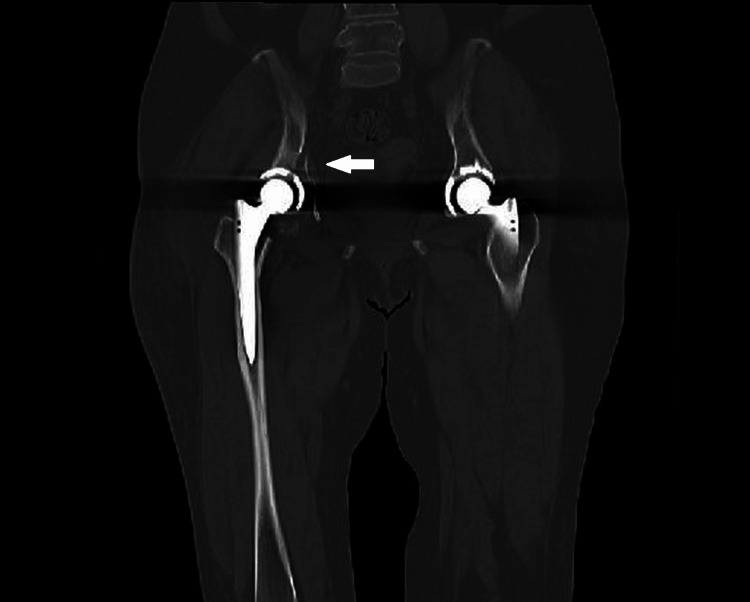
Pelvic coronal CT image at initial presentation. No clear evidence of loosening of the cup or stem was observed.

A 60-year-old woman presented with right hip pain one month after bilateral THA was performed for osteonecrosis of the femoral head. Imaging studies revealed fractures of the T-shaped acetabulum. Conservative treatment was initially selected with the plan to revise the cup once the fracture had healed (Figures 7-9).

**Figure 10:**
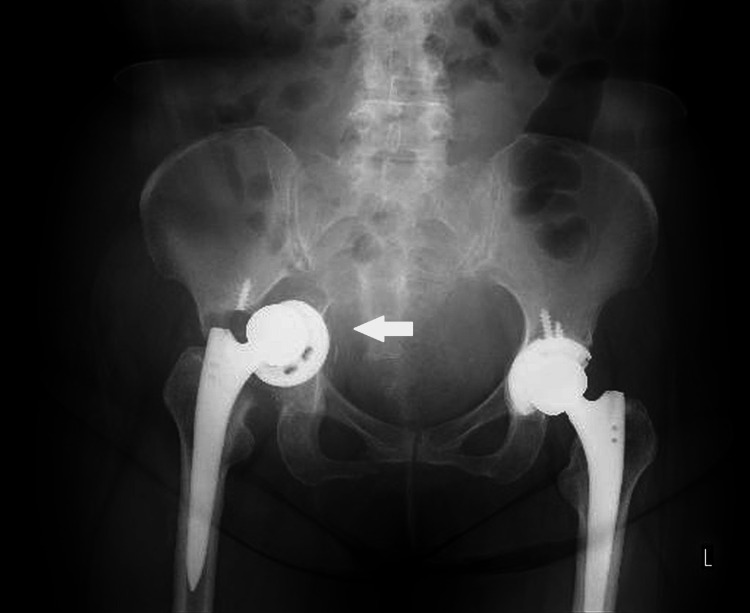
Plain radiograph of the pelvis after the secondary fall (anteroposterior view). Progressive displacement of the fracture was evident (arrow), with central dislocation of the femoral head and breakage of the acetabular cup screws.

**Figure 11:**
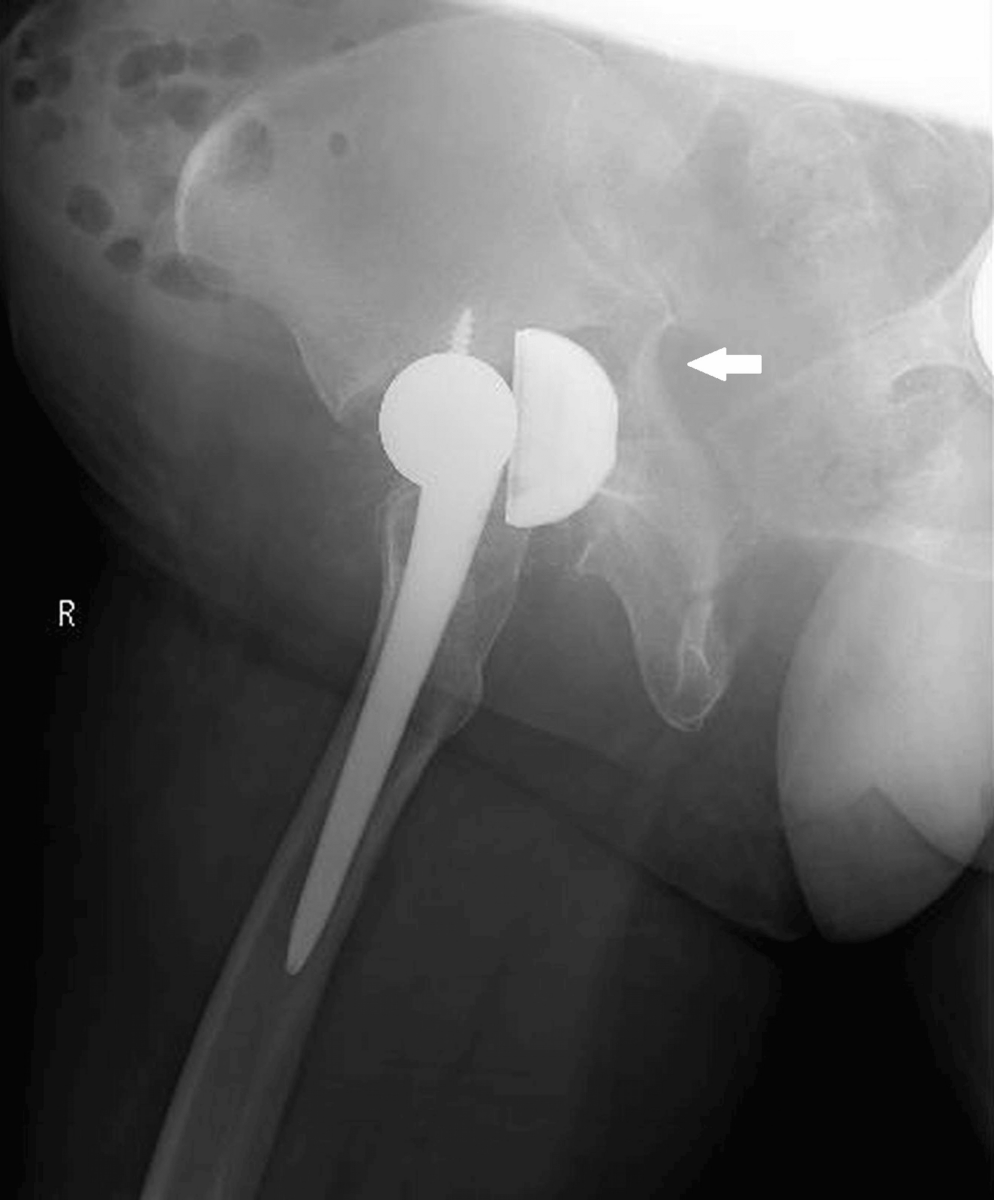
Plain radiograph of the pelvis after the secondary fall (lateral view). The fracture displacement had progressed (arrow), and the acetabular cup had become dislodged.

**Figure 12:**
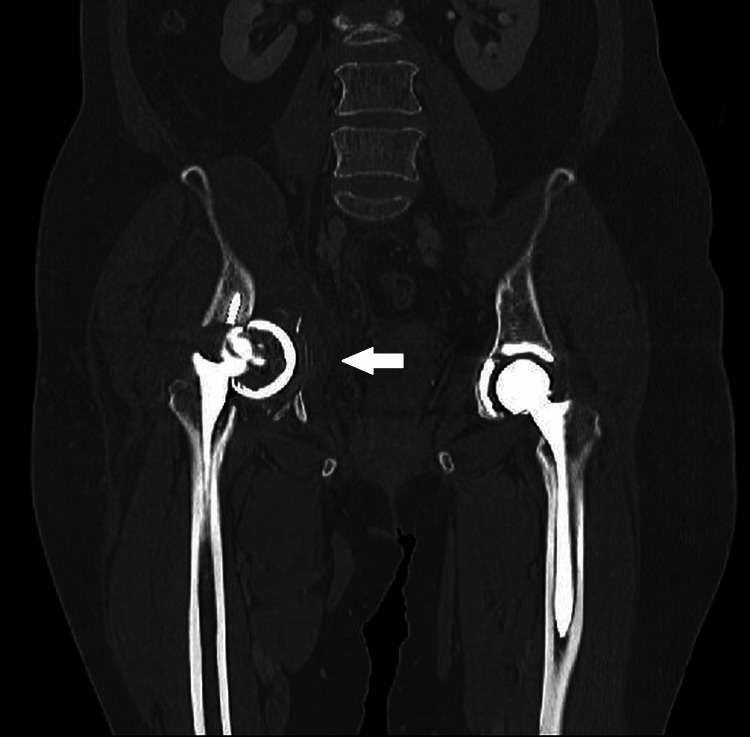
Pelvic coronal CT image after the secondary fall. There was increased displacement at the fracture site (arrow), accompanied by central dislocation. The acetabular cup was dislodged, and the screws were fractured.

However, the fracture showed progressive displacement and instability. Two months later, after another fall, further displacement and screw breakage of the acetabular component were noted, prompting surgical intervention (Figures 10-12).

**Figure 13:**
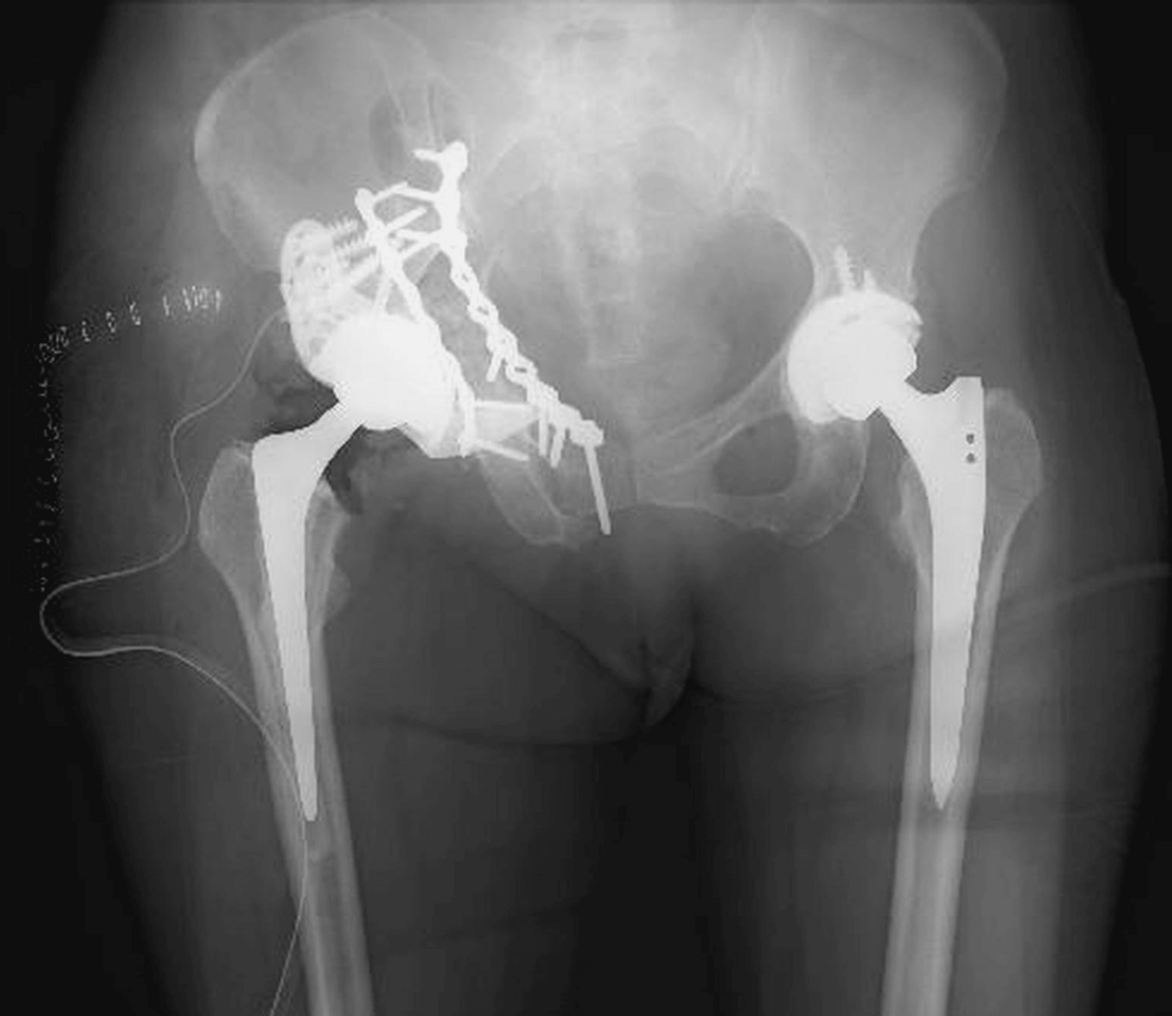
Postoperative plain radiograph of the pelvis (anteroposterior view). Plate fixation was performed from both the anterior and posterior aspects. The acetabulum was reconstructed using a cage, and the femoral stem was revised with a cemented stem.

**Figure 14:**
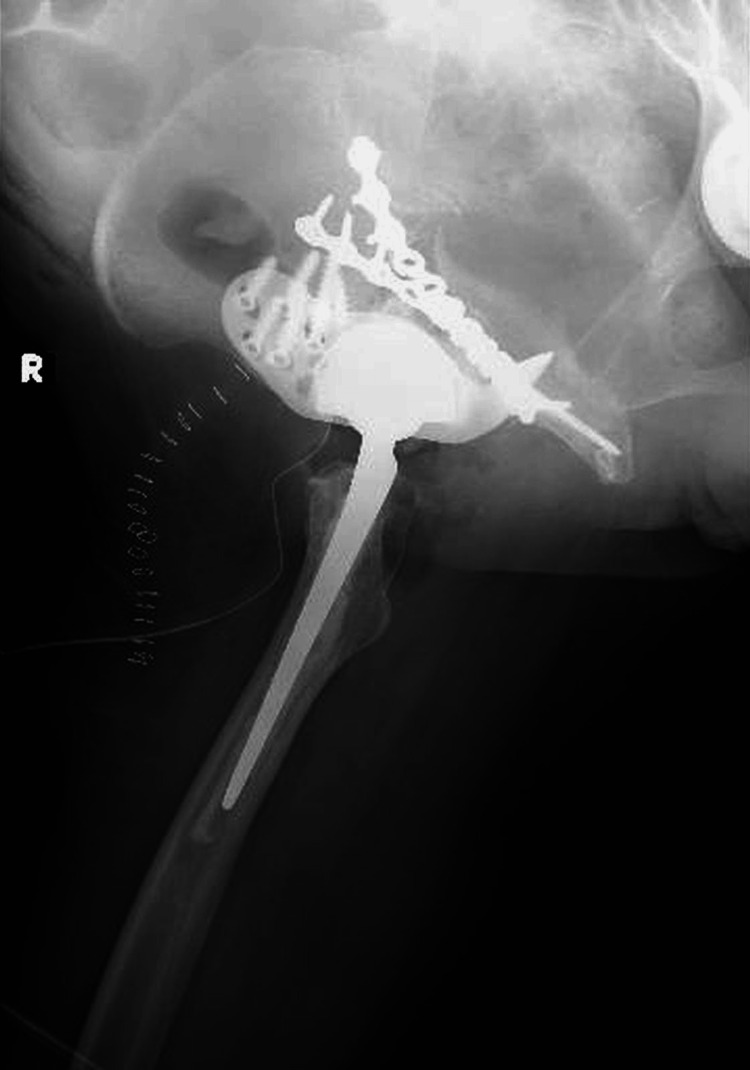
Postoperative plain radiograph of the pelvis (lateral view). Plate fixation was performed from both the anterior and posterior aspects. The acetabulum was reconstructed using a cage, and the femoral stem was revised with a cemented stem.

Surgery was performed in the supine position via a modified Stoppa approach with locking plate fixation. Due to extensive scarring and osteoporotic bone, surgical exposure was challenging. The hip was then re-approached in the lateral decubitus position using the Kocher-Langenbeck approach. The femoral head, acetabular cup, and stem were sequentially removed. Although the anterior column was stabilized with a locking plate, the posterior column remained unstable. Two locking plates were applied to the posterior column for stabilization. Acetabular reconstruction was performed using a Burch-Schneider cage (Zimmer Biomet, Warsaw, IN, USA), followed by the implantation of a cemented acetabular cup and stem (Figures 13, 14).

Postoperative rehabilitation followed the same protocol as in Case 1, with NWB initiated on the first postoperative day, PWB at four weeks, and independent ambulation achieved at three months.

## Discussion

PPAFs are a rare but significant complication that can occur following THA or BHA, with an estimated incidence of 0.07% [3,5]. Although periprosthetic femoral fractures have been widely studied, PPAF remain underreported. However, their frequency is expected to increase in the coming years, highlighting the need for accurate classification and appropriate treatment strategies [6].

PPAFs are classified based on the fracture location and implant stability. The first classification, proposed by Peterson and Lewallen, divided fractures into Type I and Type II based on acetabular cup stability [7]. The most widely used system today is the comprehensive classification by Paprosky and Della Valle (2003), which covers all known variations of PPAF, including intraoperative fractures during cup insertion or removal, traumatic and spontaneous fractures, and pelvic discontinuity. This classification also provides guidelines for both surgical and non-surgical management [3].

In the present study, both cases were categorized as Type III fractures according to this system. Case 1 was classified as Type IIIA, where acetabular component stability was maintained, and the fracture was treated with ORIF along with cup reimplantation. Case 2 initially presented as Type IIIA and was managed conservatively; however, a subsequent fall during the healing phase resulted in progression to Type IIIB with component instability, necessitating revision arthroplasty using a cage construct.

Treatment strategies for PPAF include conservative management, plate fixation, and implant revision. Conservative treatment may be considered when the implant remains stable and the fracture is minor; however, it is often limited by challenges in pain control, delayed union, and the risk of secondary fracture [7]. Plate fixation is indicated when the implant is stable but the fracture is unstable, allowing for direct anatomical reduction. The use of locking plates has demonstrated reliable fixation even in osteoporotic bone [2]. Nevertheless, prolonged weight-bearing restrictions after surgery may result in muscle weakness, necessitating well-coordinated rehabilitation.

Implant revision is reserved for cases with severe loss of acetabular component stability. These procedures often involve the use of large acetabular components or reconstruction cages, sometimes requiring structural bone grafting [8,9]. However, surgical invasiveness, particularly in elderly patients, and the limited long-term data on revision implant outcomes remain ongoing concerns.

PPAFs are also associated with a high risk of postoperative complications, including infection, heterotopic ossification, dislocation, and non-union. Selmene et al. reported dislocation in 30% and infection in 20% of cases following surgical treatment [7]. In our two cases, careful preoperative planning, appropriate surgical decision-making, and meticulous postoperative management enabled us to avoid these complications. Nevertheless, long-term follow-up is necessary to assess delayed complications such as recurrent fracture and implant loosening.

In our study, Case 1 involved a PPAF after BHA, while Case 2 occurred after THA. The former underwent early ORIF without the need for stem revision. In contrast, the latter was initially managed non-operatively, but following a second fall with increased displacement, ORIF with acetabular reconstruction using a cage and femoral stem revision was performed two months after injury. Intraoperatively, the procedure was prolonged due to extensive scar tissue and increased bleeding.

In Japan, procedures such as BHA and THA are typically performed by hip surgeons, and due to limited experience with acetabular fractures, conservative management tends to be favored. However, PPAFs generally require surgical intervention, as conservative treatment frequently fails [10]. Early surgical management may be the most effective strategy to avoid poor outcomes [7]. Peterson et al. reported that 75% of Type IIIA fractures eventually required revision surgery [2], and even if the cup appears stable immediately after the fracture, the mechanical stress of weight-bearing can lead to progressive loosening [11].

Minimally invasive plate fixation in the early phase may help avoid the need for more extensive revision. Therefore, collaboration between hip surgeons and orthopedic trauma surgeons is essential to ensure timely and appropriate management of PPAF.

Surgical treatment of acetabular fractures following BHA and THA should be based on fracture classification and implant stability, with a choice among conservative management, plate fixation, and revision arthroplasty. However, early surgical intervention generally offers favorable outcomes. Further accumulation of cases is necessary to optimize treatment strategies and evaluate long-term clinical outcomes in patients with PPAF.

Limitations

This report demonstrates certain advantages of surgical treatment for PPAF; however, several limitations must be acknowledged. First, as this is a case report involving only two patients, further prospective studies with larger cohorts are needed to evaluate the long-term efficacy and reproducibility of the proposed strategies. Second, not all PPAFs necessarily require surgical intervention. Careful patient selection is essential to maximize surgical benefits. Third, the management of PPAF often requires collaboration between hip arthroplasty specialists and trauma surgeons, which may limit the generalizability of these strategies to specialized centers equipped with both teams. Fourth, this report lacks objective outcome measures, such as validated functional scores or range of motion data. Fifth, the short follow-up duration precludes evaluation of implant longevity and late complications. Lastly, due to the urgent nature of the surgeries, no osteoporosis-specific interventions were implemented. Future follow-up studies incorporating these elements are planned to provide a more comprehensive assessment of surgical outcomes.

## Conclusions

Surgical management of PPAF following BHA or THA should be selected based on fracture type and implant stability using classification systems such as the modified Paprosky-Della Valle classification. While some cases may be managed conservatively, early surgical intervention generally yields better outcomes. Collaboration between trauma and hip surgeons is essential for optimal treatment. Further case accumulation and long-term follow-up are necessary to evaluate treatment efficacy and establish optimal strategies for managing PPAF.
